# Comparative Analysis of the National Fatality Rate in Construction Industry Using Time-Series Approach and Equivalent Evaluation Conditions

**DOI:** 10.3390/ijerph19042312

**Published:** 2022-02-17

**Authors:** Yukyung Shim, Jaemin Jeong, Jaewook Jeong, Jaehyun Lee, Yongwoo Kim

**Affiliations:** 1 Department of Safety Engineering, Seoul National University of Science and Technology, 232 Gongneung-ro, Nowon-gu, Seoul 01811, Korea; mancy99@seoultech.ac.kr (Y.S.); ss96011@seoultech.ac.kr (J.J.); archi0528@seoultech.ac.kr (J.L.); 2 Department of Construction Management, University of Washington, 105 Arch Hall, Seattle, WA 98195, USA; yongkim@uw.edu

**Keywords:** fatality rate, risk level, full-time equivalent workers, equivalent evaluation conditions, time-series analysis

## Abstract

Fatality rates such as fatalities per full-time equivalent workers are officially used to compare the risk level of the construction industry among various countries. However, each country evaluates the fatality rate using different conditions. This paper presents the comparison of fatality rates of various countries using conventional (national data) and pair (equivalent condition) methods through a time-series approach. The research was conducted in three stages. The risk level was evaluated in order in South Korea (1.54), Japan (0.84), Mexico (0.83), China (0.70), United Kingdom (0.15), and Singapore (0.13) in terms of national data. However, the risk level was re-evaluated in order in China (2.27), South Korea (2.05), Mexico (1.23), Singapore (0.98), Japan (0.80), and United Kingdom (0.47) in terms of equivalent conditions. The risk level of each can be changed when the fatality rate is compared under given equivalent conditions.

## 1. Introduction

According to the International Labor Organization (ILO), approximately 2.8 million people every year become victims of industrial disasters, occupational accidents, and occupational diseases, causing severe social and economic problems [1,2]. Over the past several decades, the construction industry has had the highest fatality rate (FR) among various industries, including the FR in industrial accidents [3,4,5,6,7,8]. Several countries implemented the Construction Design and Management (CDM), Design for Safety (DFS), and Prevent to Design to reduce the fatal accident and injury rates in the design phase in the construction industry [9,10,11,12,13,14]. In particular, in 1994, after implementing the CDM in the design phase, the fatality rate (FR) was decreased by over 40% in the United Kingdom [15].

Various indices are used to indicate the risk level of each country, such as the fatal occupational incident rate per 100,000 persons, converted accident ratio, severity rate, and incident rate [16,17,18,19,20,21]. The fatality rate (FR) is one of the probabilistic approaches, which is used to analyze the national risk level considering the number of fatal accidents and the number of workers. Because the method used to calculate the FR is relatively simple, the FR is used to compare the risk level by country [21,22,23,24,25,26,27,28]. South Korea developed and uses an evaluation index called the fatality rate per 10,000 full-time equivalent workers (FRFEW) based on the fatal occupational injuries per 100,000 workers [29]. According to the ILO, South Korea has a very high FR compared with other developed countries, and it is more than ten times higher than the FR in the United Kingdom, which has the lowest rate [21,24,25,26,27,28].

Frequently, the number of fatal accidents and full-time equivalent workers are used to calculate the FR. However, according to the ILO, each country has different methods of tallying the number of fatal accidents and full-time equivalent workers [21]. For example, in South Korea, the number of fatal accidents and the number of workers are calculated based on the data researched through the industrial accident compensation insurance [30]. However, in the United Kingdom, the number of fatal accidents is calculated based on a total inspection, and the number of workers is estimated through a sampling inspection [31,32]. Therefore, in the studies using national data, only simple comparisons of statistical industrial accident data between countries were provided, and the direct comparison between the countries was impossible [24,25,26,27,28].

Some studies compared the FR between countries to indicate the risk level. Leung and Chow (2002) used the national data of Japan, Singapore, South Korea, Taiwan, and the Philippines to compare the accident rates and policies of Southeast Asian countries [24]. Kim et al., (2010) compared the relevance of the economic indices and industrial accident indicators of ILO labor statistics databases (LABORSTA) from 1975 to 2006 for 30 member countries of the Organization for Economic Co-operation and Development (OECD) [25]. Choi et al., (2019) used the accident data of the construction industry in China, South Korea, and the United States from 2011 to 2015 to compare the similarities and differences in the mortality risk of the construction industry between the countries. The construction industries in China, South Korea, and the United States continued to have high accident probabilities, and the most common accident types were falls and crashes [26]. Choi (2020) compared the FR of 36 OECD member countries based on the data of ILOSTAT. They compared all the industrial accidents, deaths, and full-time equivalent workers in all the industries and the construction industry for 2017. The comparison results indicated that the FR of the construction industry was relatively higher than all other industries in South Korea compared with other countries [27].

Nevertheless, previous studies compared the FR between countries based on the national data and did not conduct the comparison under equivalent conditions. Because each country has a different method of tallying the number of fatal accidents and number of workers to calculate the FR, the variables used to calculate the FR must be calculated under equivalent conditions. Therefore, the variables calculated under equivalent conditions can be used to obtain the indices for the evaluation of the risk level of each country and to facilitate a fair comparative analysis.

In this study, we used a time-series approach to compare the FR based on the national data and equivalent conditions and propose the requirement for risk level assessment in each country under the equivalent conditions. The main finding of this paper reveals the differences in the investigation methods for the number of fatal accidents and the number of workers by country, and the results can be used to establish guidelines for accident investigation for each country.

## 2. Investigation of the Fatality Rate of Various Countries

In this study, the target countries selected for comparison with the FR between national data and equivalent conditions by country were (i) South Korea, (ii) Japan, (iii) China, (iv) Singapore, (v) Mexico, and (vi) the United Kingdom.

The criteria for the selection of the target countries to proceed with this study were as follows. First, the countries were selected according to the risk level based on the FR in each country by referring to the ILO and previous studies. We chose South Korea with the highest FR, the United Kingdom with the lowest FR, Japan and Mexico with a similar FR, and China and Singapore. Second, the United Kingdom (CDM), Singapore (DFS), and South Korea (DFS) implement safety management in the design phase separately to reduce or eliminate accidents and disasters in the construction industry [9,10,11]. However, Japan, China, and Mexico have no separate safety management measures in the design phase. Therefore, the FR can be compared according to the availability of safety management in the design phase.

Therefore, this study compared the FR based on the national data and equivalent conditions for six countries: South Korea, Japan, China, Singapore, Mexico, and the United Kingdom.

### 2.1. South Korea

South Korea enacted the Industrial Safety and Health Act under the Ministry of Employment and Labor in 1981 to prevent industrial accidents and create pleasant work environments with the aim of maintaining and improving the safety and health of workers [33]. The Industrial Safety and Health Act related to the construction industry includes contents such as the appointment of safety managers, the appropriation of industrial safety management budget, and the preparation and submission of hazard and risk prevention plans [33]. Furthermore, according to the Construction Technology Promotion Act of the Ministry of Land, Infrastructure and Transport (MLIT), the DFS has been enforced since 2016 to include safety in the design phase [11]. Currently, the compulsory application targets of the DFS only include those subject to traditional separate orders for design and construction and the preparation of safety management plans among public constructions, and the MLIT is planning to expand them gradually in the future [11].

In South Korea, the number of fatal accidents used to calculate the FR is provided according to two types: fatal occupational accidents and fatal occupation diseases [29]. The number of fatal accidents in South Korea is calculated through a total inspection based on the amount paid out for industrial accident compensation insurance [29,30]. Furthermore, the actual number of workers is difficult to calculate due to the number of day laborers and individual laborers who work in the construction industry. Therefore, it is calculated using the number of full-time equivalent workers according to the relevant law of South Korea. Every year, the number of workers is estimated by calculating the number of full-time equivalent workers for each construction site [29,34].

### 2.2. Japan

Japan’s Industrial Safety and Health Act stipulates the appointment of a specific general contractor for general management at construction and shipbuilding sites in which several companies operate in the same place [35]. In Japan, if a labor accident occurs, social responsibility, as well as a legal punishment, administrative penalty, and civil lawsuit, is imposed. Furthermore, based on the Act on Promotion of Housing Quality Assurance enacted in 2000, the housing performance labeling system has been implemented, and comprehensive performance assessments are conducted for new and existing buildings [36].

In Japan, the Ministry of Health, Labor and Welfare (MHLW) provides data on the number of fatal accidents and number of fatal accidents and injuries per 1000 workers in the construction industry [37]. The number of fatal accidents in Japan is counted through a total inspection, which is conducted by reporting to the administrative agency immediately after the occurrence of a fatal accident [38]. Furthermore, the number of workers used to calculate the FR is provided in terms of the numbers of employed, full-time, and temporary workers [39]. Here, the FR provided by the Ministry of Health, Labor and Welfare includes the number of full-time and temporary workers [37].

### 2.3. China

The government of China has enacted and revised several laws for environmental protection and sustainable development over the last several years. Examples include the Construction Act, Safe Production Act, Environmental Protection Act, and Urban and Rural Planning Act [40]. Moreover, the Regulations on Safety Production Management of Construction Projects was enacted in 2014 [41]. These laws and regulations recommend reducing the pace of construction in the planning, design, and construction stages of construction projects and performing strategic environmental assessments [42].

However, in China, the number of fatal accidents in the construction industry has surpassed that in the mining industry since 2012, ranking the first among all industrial and production sectors in China [8]. Furthermore, the average number of fatal accidents in the construction industry in China has exceeded 1800 and increased to more than 3800 in 2016 and 2017, which was significantly higher than the figures of many developed countries [43,44,45]. Particularly, 1732 accidents occurred, killing 1752 persons in the first half of 2018 alone [46].

The number of fatal accidents used for the FR calculation is investigated through a total inspection, and accidents must be reported immediately according to the relevant regulations [40,41]. The number of workers is collected through a sampling inspection, and the corrected results are provided. The information on the number of workers is available in the China Statistical Yearbook and the China Labor Statistical Yearbook [47].

### 2.4. Singapore

Singapore implemented the Guidelines on DFS for Building and Structures in 2008 under the influence of the CDM 94 of the United Kingdom. In 2010, the DFS Coordinator Course was implemented to recommend and support the DFS, and in 2014, the DFS was mandated [10]. In 2015, Workplace Safety and Health regulations were implemented, clearly institutionalizing the DFS. Singapore’s DFS is applied to constructions of more than SGD 100,000 (Singapore Dollar) in total cost, and it is also applied to the remodeling of applicable buildings [10]. Furthermore, the Buildability Legislations were implemented in 2010 to reduce the labor dependency on foreign workers and increase productivity in all industries [48].

Singapore provides the number of fatal accidents and the fatal incident rate per 10,000 persons every year. The number of fatal accidents used to calculate the FR is investigated through a total inspection of the workplaces and industries in which fatal accidents have occurred [49]. The number of workers in Singapore is estimated using a sampling inspection through the Labor Force Survey (LFS). The LFS targets individual households and is conducted quarterly by randomly selected respondents [50]. It excludes workers living in construction sites, dormitories, and employee residences, and people commuting from foreign countries to Singapore, and the total labor force is estimated by combining the data on the residents obtained from the survey and the non-resident employment data from administrative records [49,50].

### 2.5. Mexico

The infrastructure industries in Mexico have different guidelines depending on the construction type, and the permit types and regulations for the construction of public facilities have been specified. Generally, the federal government has authority over some laws and regulations, but it is important that each state applies its own official construction regulations [51,52].

Mexico provides information on occupational accidents and diseases through annual reports. The number of fatal accidents for the FR calculation is investigated through a total inspection, and an accident is required to be reported to the Secretariat of Labor and Social Welfare within 72 h of it occurring. The number of workers is calculated through a sampling inspection through the National Survey on Occupation and Employment (ENOE) [53,54]. However, the number of workers used for the FR calculation is estimated only using the number of insurance subscribers [55].

### 2.6. The United Kingdom

In 1974, the United Kingdom enacted the Health and Safety at Work Act and established the Health and Safety Executive (HSE) for the execution of relevant laws [56]. Furthermore, in 1994, the Construction Design and Management (CDM) was introduced, which was only applied to the construction industry, unlike the Health and Safety at Work Act, which includes all industries, and it was revised twice in 2007 and 2015. The CDM 2015 is based on the premise that safety in the construction industry must be ensured in all construction processes including the use and maintenance stages. The applicable targets are specified as projects that last longer than 30 working days and have more than 20 workers working simultaneously at any point on the project, or projects exceeding 500 man-days annually. Thus, the projects subject to the regulations include almost all constructions except small self-constructions. [9]. The effect of the adoption of the CDM can be determined from various indicators. For example, in the six years after its enforcement in 1994, the number of construction orders increased more than 1.5 times compared with the past, but the fatal incident rate per 100,000 workers decreased by approximately 40% [15].

In the United Kingdom, the HSE announces the number of fatal accidents for the FR calculation. Members of the public are excluded from the number of fatal accidents used to calculate the fatal incident rate per 100,000 workers [31,57]. The number of workers is calculated using a sampling inspection through the Annual Population Survey. The Annual Population Survey is a quarterly survey of household statistics conducted targeting randomly selected households in the United Kingdom by the Office for National Statistics (ONS). According to the ONS, the number of workers is counted targeting the employed people [32].

### 2.7. Comparison of the National Fatality Rate Data for Various Countries

As discussed above, the method of calculating the number of fatal accidents and the number of workers for FR calculation varies by country. Table 1 shows the calculation standards for the number of fatal accidents and number of workers in each country. First, the number of fatal accidents in each country is calculated through a total inspection. However, South Korea conducts the total inspection based on the industrial accident compensation insurance, and although other countries conduct the total inspection based immediate reporting to administrative agencies, some differences are observed. Second, in South Korea, the number of workers is counted by collecting the results calculated for each site using the calculation method of full-time equivalent workers, but in other countries, it is calculated through sampling inspections. However, even for sampling inspection, Singapore conducts the sampling inspection of individual households through the LFS, whereas in the United Kingdom, the ONS conducts its quarterly survey by targeting randomly selected households.

Therefore, the method of calculating the number of fatal accidents and number of workers for the FR calculation varies by country. Some previous studies indicated that the direct comparison of countries is impossible even though simple comparisons are possible because the collecting method of statistical data for industrial accidents varies by country [19,20,21,22,23]. Therefore, this study compared the FR based on the national data and equivalent conditions and recommended the requirement for risk level assessment in each country under the equivalent evaluation condition.

## 3. Materials and Methods

**Figure 1:**
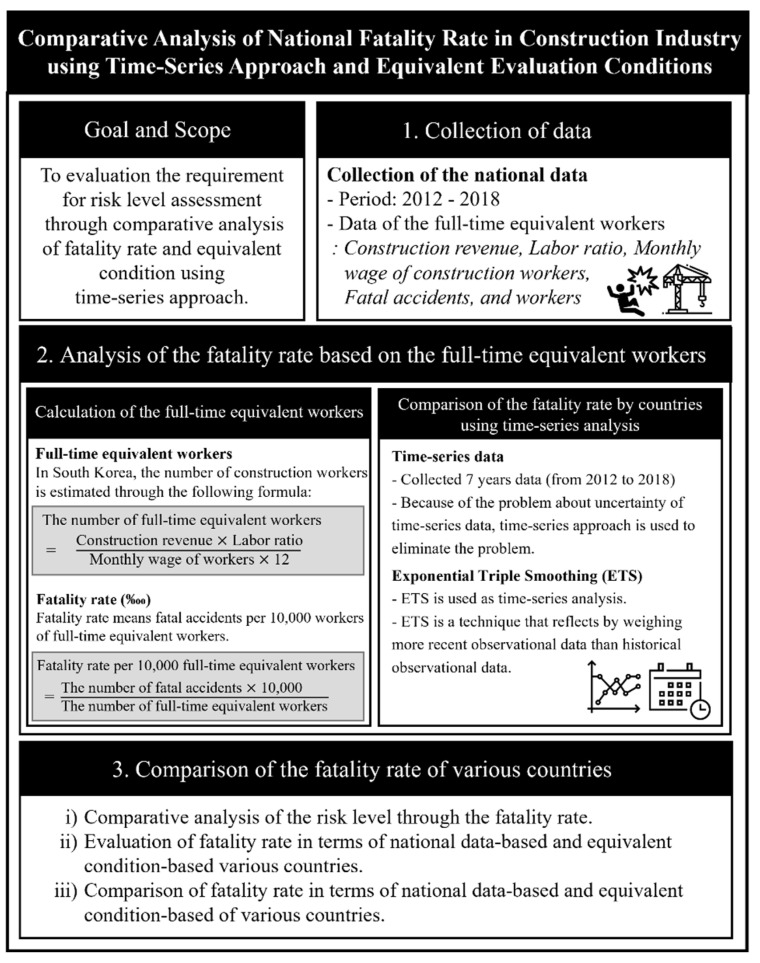
Research process.

This study was conducted in three stages, as shown in Figure 1: (1) the collection of data; (2) analysis of the fatality rate based on the full-time equivalent workers; (3) comparison of the fatality rate by countries.

In the first stage, national data were collected to calculate the FR for South Korea, Japan, China, Singapore, Mexico, and the United Kingdom. Furthermore, information such as the construction revenue, labor ratio, and the monthly wage of construction workers was collected for each country to calculate the full-time equivalent workers. In the second stage, the number of full-time equivalent workers was estimated to compare the FR of each country under the equivalent conditions. Subsequently, a time-series analysis was used to calculate the FR of each country. In the third stage, the FR was compared in terms of the national data and equivalent conditions, and the requirement for risk level assessment in each country under the equivalent conditions was presented.

### 3.1. Collection of Data

As shown in Table 2, relevant data were collected to evaluate and compare the FR based on the national data and equivalent conditions between the countries.

In this study, we collected data from 2012 to 2018. In South Korea, the classification standards of fatal accidents were revised in 2012, making them difficult to compare with previous data after 2011 [29]. In Japan, the number of fatal accidents was abnormally high in 2011 because of the Great East Japan Earthquake [37]. Therefore, in this study, we collected seven-year data from 2012 to 2018 considering the information provided by each country, and the collected data included the number of fatal accidents, number of full-time equivalent workers, construction revenue, labor ratio, and the monthly wage of construction workers in each country [29,31,32,37,39,47,49,50,55,57,58,59,60,61,62,63,64,65,66]. The data collection sources are presented in Table 2. The collected data are included in the supplementary materials (refer to the Supplementary Materials in Tables S1–S3).

### 3.2. Analysis of the Fatality Rate Based on the Full-Time Equivalent Workers

#### 3.2.1. Calculation of the Full-Time Equivalent Workers

As mentioned above, each country uses different standards to estimate the number of fatal accidents and the number of workers to calculate the FR. Regarding the number of fatal accidents, although each country uses a different data collection method, they all tally the number of fatal accidents through total inspections. However, most countries tally the number of workers through sampling inspections. Because the sampling inspection method varies by country, we cannot conclude that the data are collected under equivalent conditions [67]. The FR calculated under different conditions can be used in simple comparisons, but a direct comparison of the risk level between countries is impossible [19,20,21,22,23].

In this study, we established standards for the calculation of the number of workers to calculate the FR in each country under equivalent conditions. In South Korea, the number of full-time equivalent workers is used as the calculation method (Equation (1)) to provide the number of workers. The number of full-time equivalent workers is a formula that estimates the number of workers per year based on the revenue of the construction industry [34]. The data of the construction revenue and monthly wage of a construction worker required to calculate the number of full-time equivalent workers were collected for each country according to Table 1. The labor ratio is currently only available in South Korea and Japan. In this study, the labor ratio of South Korea was used since the number of full-time equivalent workers in South Korea was used. The data of labor ratio is included in the supplementary materials (refer to the Supplementary Materials in Table S4).

(1) $$The\;number\;of\;full-time\;equivalent\;workers=\frac{Construction\;revenue\times Labor\;ratio}{Monthly\;wage\;of\;construction\;workers\times 12}\;$$

Various formulas can be used to evaluate the risk level. Typical examples include the fatal occupational incident rate per 100,000, injury severity rate, and the incident rate [23,24,25,26,27,28]. South Korea uses the fatality rate per 10,000 full-time equivalent workers (FRFEW) as a national standard [29,30,34]. This study used the FRFEW used in South Korea to compare the FR between the countries under equivalent conditions.

The FRFEW is calculated using Equation (2) using the number of fatal accidents and number of full-time equivalent workers.

(2) $$\begin{array}{c} \mathrm{Fatality}\;\mathrm{rate}\;\mathrm{per}\;10,000\;\mathrm{full}-\mathrm{time}\;\mathrm{equivalent}\;\mathrm{workers}\;\left(‱\right)\\ =\frac{The\;number\;of\;fatal\;accidents}{The\;number\;of\;full-time\;equivalent\;workers}\times 10,000 \end{array}$$

#### 3.2.2. Comparison of the Fatality Rate by Countries Using Time-Series Analysis

In this study, the time-series data from 2012 to 2018 was collected. Because uncertainty is inherent in time-series data, the uncertainty problem is difficult to resolve through the averages. Time-series analysis can resolve the uncertainty problem by applying regular patterns of the time-series data containing the uncertainty [68]. In this study, we applied exponential triple smoothing (ETS) to analyze the irregularities in the time-series data. ETS is a technique that reflects the latest trend by assigning more weights to recent observation data than past observation data. ETS is performed using Equations (3)–(6) [69]. In this study, national data of seven years from 2012 to 2018 were collected, and the national-data-based FRFEW and equivalent-condition-based FRFEW were comparatively analyzed using ETS. The time-series analysis is calculated by using Data analysis, which is an add-on in Excel 2016.

(3) $$S_{t}=\alpha \frac{y_{t}}{I_{t-L}}+\left(1-\alpha \right)\left(S_{t-1}+b_{t-1}\right)$$

(4) $$b_{t}=\gamma \left(S_{t}-S_{t-1}\right)+\left(1-\gamma \right)b_{t-1}$$

(5) $$I_{t}=\beta \frac{y_{t}}{S_{t}}+\left(1-\beta \right)I_{t-L}$$

(6) $$F_{t+m}=\left(S_{t}+mb_{t}\right)I_{t-L+m}$$

where *y* is the observation of the time-series approach, *S* is the smoothed observation of the time-series approach, *b* is the trend factor, *I* is the seasonal factor, *F* is the forecast at period m, *t* is a time period, α is the constant for *S_t_*, *β* is the constant for *b_t_*, and γ is the constant for *I_t_*.

### 3.3. Normalization of the Fatality Rate under National Data and Equivalent Conditions

In the previous chapters, using the historical data and time-series approach, the FRFEW was calculated under national data and equivalent conditions. The FRFEW presents the fatal accident per the full-time equivalent workers as a probabilistic approach. However, since the characteristics of construction by each country is different, such as the major type of facility, construction methods, and accident types, the probabilistic approach might not present the risk level reflected by the characteristic of construction [70]. Thus, normalization is used to present equivalent conditions from different points of view. By means of normalization, the value is converted ranging from 0 to 1. A value of 1 indicates the highest number of fatal accidents or the highest number of full-time equivalent workers in each country [71]. The normalized fatality rate is calculated by Equations (7)–(9). The normalized fatality rate (NFR) is calculated as the number of fatal accidents divided into the number of full-time equivalent workers. Through NFR, the risk level can be evaluated by reflecting the level of the number of fatal accidents and full-time equivalent workers based on historical data. The NFR is calculated under national data and equivalent conditions.

(7) $$Normalized\;fatal\;accident_{i}=\frac{Fatal\;accident_{i}-Fatal\;accident_{i}^{Max}}{Fatal\;accident_{i}^{Max}-Fatal\;accident_{i}^{Min}}$$

(8) $$\begin{array}{l} Normalized\;full-time\;equivalent\;worekrs_{i}\\ =\frac{Full-time\;equivalent\;worekrs_{i}-Full-time\;equivalent\;worekrs_{i}^{Max}}{Full-time\;equivalent\;worekrs_{i}^{Max}-Full-time\;equivalent\;worekrs_{i}^{Min}} \end{array}$$

(9) $$Nomalized\;fatality\;rate_{i}=\frac{Normalized\;fatal\;accident_{i}}{Normalized\;full-time\;equivalent\;workers_{i}}\;$$

where *fatal accident_i_* is the i-th value of the number of fatal accidents for historical data and estimation values, and *full-time equivalent worker_i_* is the i-th the number of full-time equivalent workers for historical data and estimation values.

### 3.4. Comparison of the Fatality Rate of Various Countries

As described above, this study collected the national data for each country and proposed the FRFEW based on the number of full-time equivalent workers to perform the comparative analysis under the equivalent condition. To assess the risk level for each country, the following analysis and comparison were performed. First, the risk level was analyzed and compared for the countries using the FRFEW of national data of each country. Second, the FRFEW under the equivalent condition was evaluated and compared for the countries. Third, the results of the FRFEW for the national data and equivalent conditions for each country were compared. Forth, the normalized results for the national data and equivalent conditions for each country were compared.

## 4. Results and Discussion

### 4.1. Comparison of the Fatality Rate in Terms of the National Data of Various Countries

Table 3 shows information on the number of fatal accidents by country from 2012 to 2018. The average number of fatal accidents in each country was 23 at the minimum and 2880 at the maximum. Table 3 indicates that the number of fatal accidents in each country fluctuates repeatedly every year.

(I) The annual number of fatal accidents in South Korea was between a minimum of 434 and a maximum of 516, with an average of 477. When compared with other countries, it was at the highest level, except for China, which had the highest number of fatal accidents.
(II) The annual number of fatal accidents in Japan was between a minimum of 294 and a maximum of 377, with an average of 334. Japan ranked third in the number of fatal accidents.
(III) The annual number of fatal accidents in China was between a minimum of 1891 and a maximum of 3843, with an average of 2880. China had the highest number of fatal accidents among the compared countries. When compared with the figure for Singapore, which had the lowest number of fatal accidents, it was approximately 125 times higher.
(IV) The annual number of fatal accidents in Singapore was between a minimum of 12 and a maximum of 34, with an average of 23. Singapore had the lowest number of fatal accidents among the compared countries.
(V) The annual number of fatal accidents in Mexico was between a minimum of 150 and a maximum of 220, with an average of 187, which ranked fourth among the compared countries.
(VI) The annual number of fatal accidents in the United Kingdom was between a minimum of 31 and a maximum of 47, with an average of 38. When compared with the figure for Singapore, it was approximately 1.5 times higher, ranking fifth among the compared countries.

**Figure 2:**
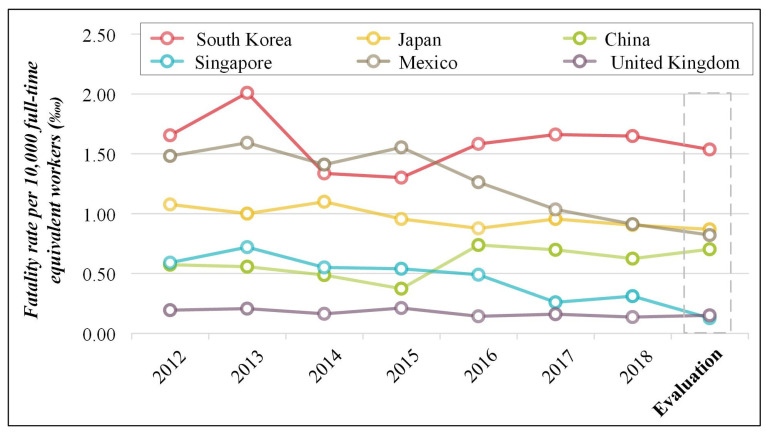
Fatality rate per 10,000 full-time equivalent workers in terms of the national data of various countries.

As shown in Table 4 and Figure 2, the FRFEW was evaluated and compared for various countries in terms of national data. When compared with Table 3, the FRFEW in each country largely fluctuated repeatedly. However, even when the highest number of fatal accidents occurred in each country, the FRFEW was not the highest. For example, China had the highest number of fatal accidents with 3843 incidents in 2017, but the highest FRFEW was 0.74‱ in 2016. Because the FRFEW has an annual uncertainty every year, it is very difficult to evaluate. Therefore, this study evaluated the FRFEW using a time-series analysis instead of the average to resolve the uncertainty.

(I) The FRFEW of South Korea was between 1.30‱ and 2.01‱, and the FRFEW based on the time-series data was 1.54‱. South Korea ranked second in the number of fatal accidents but had the highest risk level from the perspective of the FRFEW.
(II) The FRFEW of Japan was between 0.88‱ and 1.10‱, and the FRFEW based on the time-series data was 0.87‱. Japan ranked second after South Korea.
(III) The FRFEW of China was between 0.37‱ and 0.74‱, and the FRFEW based on the time-series data was 0.71‱. Compared with other countries, China had the highest number of fatal accidents. However, when the number of workers provided in Table S1 was considered, the number was up to 100 times higher than that of other countries. Therefore, although the number of fatal accidents was large, the risk level was lower than that of other countries if the number of workers was considered in the comparison.
(IV) The FRFEW of Singapore was between 0.26‱ and 0.72‱, and the FRFEW based on the time-series data was 0.13‱. Singapore had the lowest number of fatal accidents and FRFEW compared with other countries.
(V) The FRFEW of Mexico was between 0.91‱ and 1.59‱, and the FRFEW based on the time-series data was 0.82‱. When compared with the number of fatal accidents, Mexico’s FRFEW indicated a higher risk level because the number of fatal accidents was high relative to the number of workers.
(VI) The FRFEW of the United Kingdom was between 0.14‱ and 0.21‱, and the FRFEW based on the time-series data was 0.15‱, indicating a higher risk level than Singapore.

### 4.2. Comparison of the Fatality Rate of Various Countries in Terms of Equivalent Conditions

**Figure 3:**
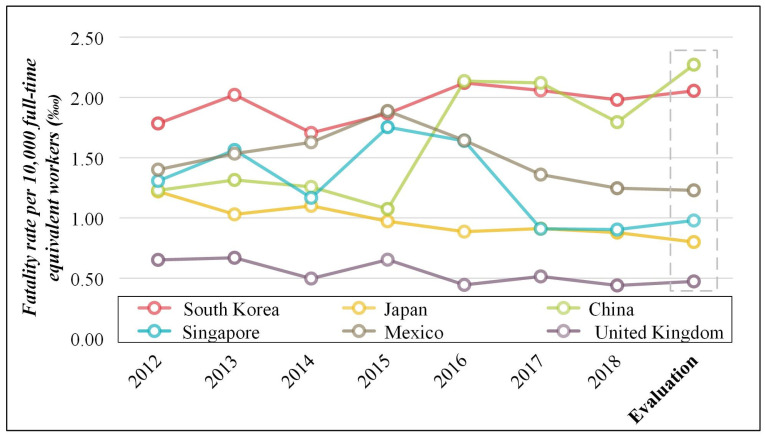
Fatality rate per 10,000 full-time equivalent workers considering the equivalent condition of various countries.

As shown in Table 5 and Figure 3, the FRFEW was evaluated and compared for various countries in terms of equivalent conditions. As shown in Table 1, the methods of tallying the number of fatal accidents and number of workers required to calculate the FRFEW differed by country. Each country tallies the number of fatal accidents through the total inspection using a different method. However, the number of workers is corrected and provided after performing a prediction or sampling inspection according to each country’s standards. Hence, a comparison under equivalent conditions with the data calculated based on different standards between the countries is difficult. Therefore, in this study, before evaluating and comparatively analyzing the FRFEW under equivalent conditions, the FRFEW for each country was calculated based on the equivalent condition presented in Section 3.2. The number of full-time equivalent workers in various countries is included in the Supplementary materials (refer to the Supplementary Materials in Table S5).

(I) In South Korea, the FRFEW was between 1.71‱ and 2.12‱, and the FRFEW based on the time-series data was 2.05‱. The number of workers in South Korea decreased compared with that in the national data, thereby increasing the FRFEW. However, unlike the result above, in which the risk level was the highest, the risk level of South Korea was the second highest.
(II) In Japan, the FRFEW was between 0.88‱ and 1.22‱, and the FRFEW based on the time-series data was 0.80‱. It was lower than the FRFEW of the national data of Japan. However, the risk level decreased.
(III) In China, the FRFEW was between 1.07‱ and 2.14‱, and the FRFEW based on the time-series data was 2.29‱. The comparison of Table S1 and Table S5 shows that the annual number of workers based on the equivalent condition decreased more than twice compared with the annual number of workers based on the national data. Thus, China had the highest FRFEW compared with other countries.
(IV) In Singapore, the FRFEW was between 0.90‱ and 1.75‱, and the FRFEW based on the time-series data was 0.98‱, indicating a large increase in the risk level compared with the national-data-based FRFEW. Thus, the risk level was higher than that of Japan and the United Kingdom.
(V) In Mexico, the FRFEW was between 1.25‱ and 1.89‱, and the FRFEW based on the time-series data was 1.23‱. Mexico’s risk level increased slightly compared with the national-data-based FRFEW above, but the risk level by country was at an equivalent level.
(VI) In the United Kingdom, the FRFEW was between 0.44‱ and 0.67‱, and the FRFEW based on the time-series data was 0.47‱. The risk level of the United Kingdom increased slightly compared with the national-data-based FRFEW, but the national risk level decreased to the lowest level.

### 4.3. Comparison of the Fatality Rate between the National Data and Equivalent Evaluation Conditions

In this study, the FRFEW was comparatively analyzed using the national data and the equivalent condition as the risk level of each country to present the requirement for equivalent evaluation conditions of the FR to evaluate the risk level of each country.

**Figure 4:**
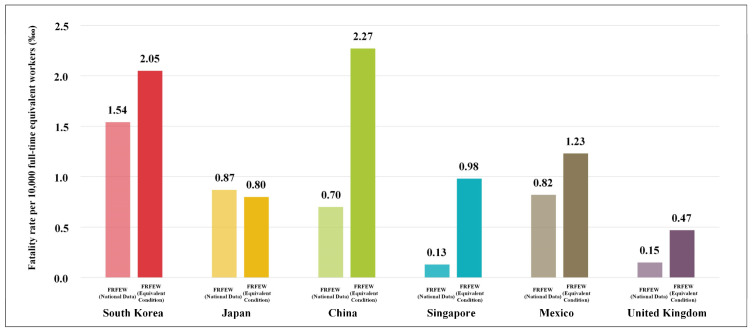
Comparison of the fatality rate per 10,000 full-time equivalent workers for national data and equivalent condition.

As shown in Figure 4, the FRFEW was evaluated in terms of national data and the equivalent condition using a time-series approach, and the results were compared. When the national-data-based FRFEW and equivalent-condition-based FRFEW were compared, the following changes were observed. First, the difference in the FRFEW between all countries changed under the equivalent condition. In terms of national data, the difference between South Korea, whose FRFEW was the highest (1.54‱), and the United Kingdom, whose was the lowest (0.15‱), was approximately ten times. However, in terms of the equivalent condition, the difference between South Korea (2.05‱) and the United Kingdom (0.47‱) was approximately four times. Accordingly, the gap between the risk level of each country decreased. The national-data-based FRFEW was the highest in South Korea (2.05‱) and the lowest in Singapore (0.13‱). However, the equivalent-condition-based FRFEW was the highest in China (2.29‱) and the lowest in the United Kingdom (0.47‱).

### 4.4. Comparison of the Normalized Fatality Rate between the National Data and Equivalent Conditions

**Figure 5:**
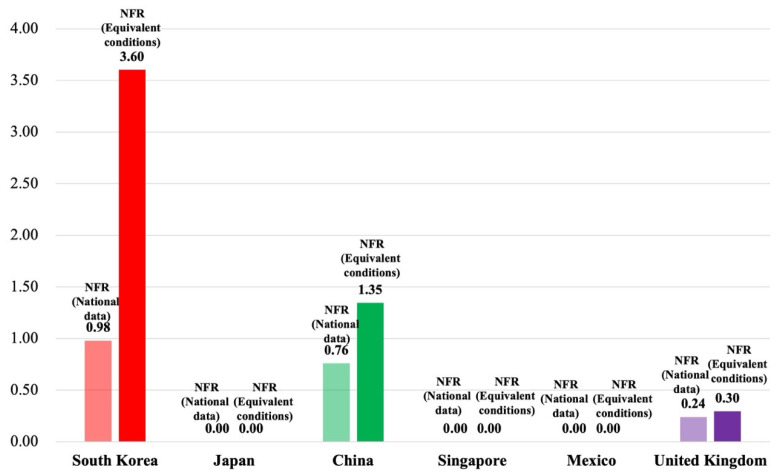
Comparison of the normalized fatality rate between national data and equivalent conditions.

As shown in Table 6 and Figure 5, the normalized fatality rate (NFR) was presented and compared between six countries under national data and equivalent conditions. In Table 6, the max value and min value are presented, which were considered by historical data and estimation value for each country. The NFR was converted by considering the estimation value for each country under national data and equivalent conditions.

In terms of the national-data-based NFR, the highest NFR was, in order, South Korea (0.98), China (0.76), and the United Kingdom (0.24). Based on the historical data and estimated value, the national-data-based NFR was reflected by characteristics of construction in accordance with the fatal accidents and full-time equivalent workers for each country. When considering the yearly number of fatal accidents compared to the yearly number of full-time equivalent workers in South Korea, China, and the United Kingdom, the risk level was not too high from the point of view of the national-data-based NFR. The national-data-based NFR was 0.00 in other countries. In calculating the normalization, the number of fata incidents or the number of full-time equivalent workers had a minimum value. Thus, the national-data-based NFR was calculated by 0.00.

In terms of the equivalent-conditions-based NFR, the highest NFR was, in order, South Korea (3.60), China (1.35), and the United Kingdom (0.30). When the equivalent-conditions-based NRF was above 1.00, the risk level could be considered high. For instance, the equivalent-condition-based NFR was 3.60 in South Korea. As the normalized fatal incident was 0.69 and the normalized full-time equivalent workers was 0.19, these values were low compared to the annual number of fatal accidents and the annual number of full-time equivalent workers from 2012 to 2018. However, as a result of the equivalent-conditions-based NFR which was divided the normalized fatal incident by the normalized full-time equivalent workers, it was confirmed that the risk level was very high compared to other countries.

### 4.5. Discussion

The FR and FRFEW based on the number of workers are probabilistic indexes to identify the risk level by country [21,22,23,24,25,26,27,28]. In this study, the FRFEW was analyzed and compared in terms of the national data and the equivalent condition to present the requirement for equivalent evaluation conditions of the FR to evaluate the risk level by country.

To evaluate the risk level by country, South Korea, Japan, China, Singapore, Mexico, and the United Kingdom were selected considering the risk level and safety management regulations. Based on this, we confirmed that the FR results of each country currently provided by the ILO differed from those under the equivalent condition. First, the analysis results of the national-data-based FRFEW in this study indicated that the difference in the FRFEW was approximately 12 times between South Korea (1.54‱), whose risk level was the highest, and Singapore (0.13‱), whose was the lowest. This implied that the risk level of the construction industry in South Korea is approximately 12 times higher than that of Singapore. However, under the equivalent condition, the FRFEW of China (2.29‱) was higher than that of South Korea (2.05‱), which had the highest risk level, and the FRFEW of the United Kingdom (0.47‱) was lower than that of Singapore (0.98‱), which had the lowest risk level. Furthermore, the difference in the risk levels between South Korea and Singapore was approximately two times in contrast to the approximately 12 times difference for the national-data-based FRFEW. This confirmed that the gap in the risk level of the construction industry decreased between the countries. Second, the risk level rankings for the six countries were South Korea (1.54‱)—Japan (0.87‱)—Mexico (0.82‱)—China (0.71‱)—the United Kingdom (0.15‱)—Singapore (0.13‱). However, under the equivalent condition, the risk level rankings changed to China (2.29‱)—South Korea (2.05‱)—Mexico (1.23‱)—Singapore (0.98‱)—Japan (0.80‱)—the United Kingdom (0.47‱). This implied that the rankings of the conventional risk levels provided by the ILO can change when the risk level is evaluated under the equivalent condition for every country.

In this study, we confirmed that the methods of tallying the number of fatal accidents and number of workers used to calculate the FR to evaluate the risk level differ by country. Some previous studies also stated that the national statistical data of industrial accidents can be used for only simple comparisons, and direct comparisons between countries are impossible [21,22,23,24,25,26,27,28]. This study, however, compared between the national-data-based FRFEW and equivalent-conditions-based FRFEW, unlike the previous studies that only presented rankings in consideration of risk level for each country. According to the ILO, the FRFEW in South Korea is 10 times higher than the FRFEW in the United Kingdom in terms of the national-data-based FRFEW [21,24,25,26,27,28]. However, when compared under equivalent conditions, the difference in FRFEW between South Korea and the United Kingdom was reduced by about 4 times (refer to Table 5). The results of this study confirmed that the risk level difference can change between countries under the equivalent condition, and the risk level rankings can be changed among the countries.

In particular, the working hours are irregular, and the proportion of temporary workers and daily workers is high. Most of their accidents are dealt with without being reported to the national institution. Thus, it is difficult to collect accurate data [23,72,73,74]. Therefore, it is necessary to prepare the standards for the national accident database before comparing the risk level by each country. This study can be used as a basis for the investigation guidelines for construction accident by each country.

To identify the risk level from a different point of view, the normalized fatality rate was analyzed under national data and equivalent conditions by each country. The normalization was calculated by using historical data and the time-series approach by each country. Thus, in consideration of the number of annual fatal accidents and full-time equivalent workers, the risk level for each country was suggested. However, it is judged that there is a problem in that the risk level may be underestimated or overestimated because normalization was carried out using historical data by each country without considering historical data from other countries. To compare the risk level by country through normalization, the risk level should be reflected by the fatal accidents, full-time equivalent workers, and characteristics of construction for other countries.

## 5. Conclusions

The study compared the fatality rate (FR) of the national data and equivalent conditions using a time-series approach to present the requirement for risk level assessment of each country under the equivalent evaluation condition.

The national-data-based FR indicated that the calculation standards differ by country. To analyze and compare the FR of each country under the equivalent condition, this study was conducted in three stages: (i) the collection of data; (ii) calculation of the full-time equivalent workers; (iii) comparison of the fatality rate of various countries.

(i) Collection of data: Data were collected to calculate the number of fatal accidents, number of workers, and the number of full-time equivalent workers in the construction industry of South Korea, Japan, China, Singapore, Mexico, and the United Kingdom.
(ii) The calculation of the full-time equivalent workers: The fatality per 10,000 full-time equivalent workers (FRFEW) was calculated based on the number of full-time equivalent workers to calculate the FR of each country under the equivalent condition. Furthermore, a time-series approach was used to resolve the uncertainty problem of the FR.
(iii) The comparison of the FR of various countries: The FRFEW was compared based on the national data and equivalent conditions. Subsequently, the requirement for the calculation of the risk level of each country under the equivalent evaluation condition was presented.

The results of the analysis and comparison of the FRFEW based on the national data and equivalent conditions are as follows: When the national-data-based FRFEW was analyzed, the country with the highest and lowest risk levels was South Korea (1.54‱) and Singapore (0.13‱), respectively. However, under the equivalent condition, the country with the highest and lowest risk levels was China (2.29‱) and the United Kingdom (0.47‱). Therefore, we confirmed that the gap between the risk level of each country under the equivalent condition decreased compared with the gap under the national-data-based risk level of each country. Furthermore, in terms of the national-data-based FRFEW, the risk level rankings of the six countries were South Korea (1.54‱)—Japan (0.87‱)—Mexico (0.82‱)—China (0.71‱)—the United Kingdom (0.15‱)—Singapore (0.13‱). However, in terms of the equivalent-condition-based FRFEW, the rankings changed to China (2.29‱)—South Korea (2.05‱)—Mexico (1.23‱)—Singapore (0.98‱)—Japan (0.80‱)—the United Kingdom (0.47‱).

The contributions of this study are as follows. From a research perspective, first, we confirmed that the tallying methods of the number of fatal accidents and number of workers used for the FR calculation differ by country. Second, the FR of each country was calculated under the equivalent condition, which was confirmed to be different under the national-data-based result. From a policy perspective, we presented the requirement to apply the equivalent evaluation condition to evaluate the FR, which is used by international organizations to assess the risk level of each country. The main finding of this paper reveals the difference in the investigation methods for the number of fatal accidents and the number of workers by country, and the results can be used to establish guidelines for accident investigation for each country.

The limitations of this study are as follows. (1) The number of full-time equivalent workers used to calculate the number of workers in this study was obtained using an estimation method instead of a full inspection, and it differed from the actual number of workers in each country. Therefore, calculating the de facto risk level of each country was difficult. (2) The labor ratio used in this study was based on the standards of South Korea and did not reflect the economic and social characteristics of other countries. (3) In this study, the probabilistic approach and normalization were used to calculate the risk level as equivalent conditions by each country. However, the characteristics of construction by each country, such as GDP from construction or the main construction facility type, were not reflected adequately. Therefore, it is hard to suggest the actual FR by each country.

In this study, the equivalent-conditions-based FRFEW was compared to six countries in the construction industry. In future studies, the FR will be expanded to OECD 38 countries and evaluated. The actual risk level will be provided by reflecting the characteristics of the construction industry by each country. Furthermore, a new method is aimed to be proposed to calculate the number of workers under the equivalent conditions, reflecting the characteristics of different countries.

## Figures and Tables

**Table 1:** Comparison of the investigation on the number of fatal accidents and full-time equivalent workers by various countries.

	South Korea	Japan	China	Singapore	Mexico	United Kingdom
Fatal accident	Total inspection (information on industrial accident compensation insurance)	Total inspection (report to the administration)	Total inspection (report to the administration)	Total inspection (report to the administration)	Total inspection (report to the administration)	Total inspection (report to the administration)
Full-time equivalent workers	Calculation of the full-time equivalent workers	Sampling inspection	Sampling inspection	Sampling inspection	Sampling inspection	Sampling inspection

**Table 2:** Data sources for collection of the national data.

Classification	South Korea	Japan	China	Singapore	Mexico	United Kingdom
Fatal accident	MOEL	MHLW	MEM	MOM	IMSS	HSE
Full-time equivalent workers	MOEL	SBJ	NBSC	MOM	IMSS	ONS
Construction revenue	KOSIS	SBJ	NBSC	DOS	INEGI	ONS
Labor ratio	MOEL	-	-	-	-	-
Monthly wage of construction workers	MOEL	MHLW	NBSC	DOS	INEGI	ONS

Note: MOEL means Ministry of Employment and Labor; KOSIS means Korean Statistical Information Service; MHLW means Ministry of Health, Labor and Welfare; SBJ means Statistics Bureau of Japan; MEM means Ministry of Emergency Management; NBSC means National Bureau of Statistics of China; MOM means Ministry of Manpower; DOS means Singapore Department of Statistics; IMSS means Mexican Institute of Social Security; INEGI means National Institute of Statistics and Geography; HSE means Health and Safety Executive; and ONS means Office for National Statistics.

**Table 3:** Analysis of the number of fatal accidents from the national data of various countries.

Year	South Korea	Japan	China	Singapore	Mexico	United Kingdom
	Fatal Accidents	Fatal Accidents	Fatal Accidents	Fatal Accidents	Fatal Accidents	Fatal Accidents
2012	461	367	2431	26	196	40
2013	516	342	2489	34	193	44
2014	434	377	2197	27	193	35
2015	437	327	1891	27	220	47
2016	499	294	3806	24	192	31
2017	506	323	3843	12	162	37
2018	485	309	3504	14	150	31
Average	477	334	2880	23	187	38
Min	434	294	1891	12	150	31
Max	516	377	3843	34	220	47
Rank	2	3	1	6	4	5

**Table 4:** Analysis of the fatality rate per 10,000 full-time equivalent workers considering national data.

Year	South Korea	Japan	China	Singapore	Mexico	United Kingdom
	FRFEW (‱)	FRFEW (‱)	FRFEW (‱)	FRFEW (‱)	FRFEW (‱)	FRFEW (‱)
2012	1.65	1.08	0.57	0.59	1.58	0.19
2013	2.01	1.00	0.56	0.72	1.59	0.21
2014	1.34	1.10	0.49	0.55	1.41	0.16
2015	1.30	0.96	0.37	0.54	1.55	0.21
2016	1.58	0.88	0.74	0.49	1.26	0.14
2017	1.66	0.96	0.70	0.26	1.04	0.16
2018	1.65	0.90	0.62	0.31	0.91	0.14
Evaluation	1.54	0.87	0.71	0.13	0.82	0.15
Min	1.30	0.88	0.37	0.26	0.91	0.14
Max	2.01	1.10	0.74	0.72	1.59	0.21
Rank	1	2	4	6	3	5

**Table 5:** Analysis of the fatality rate per 10,000 full-time equivalent workers considering equivalent conditions.

Year	South Korea	Japan	China	Singapore	Mexico	United Kingdom
	FRFEW (‱)	FRFEW (‱)	FRFEW (‱)	FRFEW (‱)	FRFEW (‱)	FRFEW (‱)
2012	1.78	1.22	1.23	1.31	1.49	0.65
2013	2.02	1.03	1.32	1.56	1.53	0.67
2014	1.71	1.1	1.26	1.17	1.63	0.5
2015	1.87	0.97	1.07	1.75	1.89	0.65
2016	2.12	0.89	2.14	1.64	1.64	0.44
2017	2.06	0.91	2.12	0.91	1.36	0.51
2018	1.98	0.88	1.82	0.9	1.25	0.44
Evaluation	2.05	0.80	2.29	0.98	1.23	0.47
Min	1.71	0.88	1.07	0.90	1.25	0.44
Max	2.12	1.22	2.14	1.75	1.89	0.67
Rank	2	5	1	4	3	6

**Table 6:** Analysis of the normalized fatality rate under national data and equivalent conditions.

National Data	Fatal Accident			Full-Time Equivalent Workers			Fatality Rate	Normalized Fatality Rate
Nation	Estimation	Max	Min	Estimation	Max	Min	Estimation (‱)	
South Korea	490	516	434	3,123,661	3,358,813	2,566,832	1.54	0.98
Japan	293	377	293	3,377,841	3,430,000	3,350,000	0.87	0.00
China	4093	4093	1891	59,404,154	55,399,100	42,439,900	0.70	0.76
Singapore	11	34	11	442,464	501,200	440,700	0.13	0.00
Mexico	145	220	145	1,707,758	1,643,363	1,211,501	0.82	0.00
United Kingdom	36	47	31	2,366,658	2,313,000	2,062,000	0.15	0.24
Equivalent Condition	Fatal Accident			Full-Time Equivalent Workers			Fatality Rate	Normalized Fatality Rate
Nation	Estimation	Max	Min	Estimation	Max	Min	Estimation (‱)	
South Korea	490	516	434	2,387,242	2,584,749	2,308,052	2.15	3.60
Japan	293	377	293	3,607,727	3,607,727	3,008,072	1.22	0.00
China	4093	4093	1891	19,175,850	19,767,905	17,462,867	2.29	1.35
Singapore	11	34	11	113,867	231,309	113,867	0.98	0.00
Mexico	145	220	145	1,186,246	1,313,237	1,165,728	1.23	0.00
United Kingdom	36	47	31	722,636	722,636	612,849	0.47	0.30

## Data Availability

The data presented in this study are available in the manuscript.

## Supplementary Materials

The following supporting information can be downloaded at: https://www.mdpi.com/article/10.3390/ijerph19042312/s1, Table S1: Analysis of the number of workers in the construction industry considering national data; Table S2: Analysis of the amount of construction revenue for calculation of full-time equivalent workers considering the equivalent conditions; Table S3: Analysis of the amount of monthly wage of construction workers for calculation of full-time equivalent workers considering the equivalent conditions; Table S4: The labor ratio of South Korea for the construction industry; Table S5: Analysis of the number of full-time equivalent workers considering the equivalent conditions by various countries.
