# Supplementary material for: Comparative Analysis of the National Fatality Rate in Construction Industry Using Time-Series Approach and Equivalent Evaluation Conditions
Source: Int J Environ Res Public Health. 2022 Feb 17;19(4):2312. doi: 10.3390/ijerph19042312 (PMC8872405; doi:10.3390/ijerph19042312)
Supplement: Supplementary file 1 [file ijerph-19-02312-s001.zip › ijerph-1565386-Supplementary.pdf]

International Journal of
Environmental Research and Public Health

MDPI

Article

Supplementary Materials for Comparative Analysis of the National Fatality Rate in Construction Industry Using Time-Series Approach and Equivalent Evaluation Conditions

Yukyung Shim [1], Jaemin Jeong [1], Jaewook Jeong [1,*], Jaehyun Lee [1] and Yongwoo Kim [2]

[1] Department of Safety Engineering, Seoul National University of Science and Technology, 232 Gongneung-ro, Nowon-gu, Seoul 01811, Korea; mancy99@seoultech.ac.kr (Y.S.); ss96011@seoultech.ac.kr (J.J.); archi0528@seoultech.ac.kr (J.L.)
[2] Department of Construction Management, University of Washington, 105 Arch Hall, Seattle, WA 98195, United States America; yongkim@uw.edu (Y.K.)
* Correspondence: jaewook.jeong@seoultech.ac.kr; Tel.: +82-2-970-6381

TABLE Captions

Table S1. Analysis of the number of workers in the construction industry considering national data

Table S2. Analysis of the amount of construction revenue for calculation of full-time equivalent workers considering the equivalent conditions

Table S3. Analysis of the amount of monthly wage of construction workers for calculation of full-time equivalent workers considering the equivalent conditions

Table S4. The labor ratio of South Korea for the construction industry

Table S5. Analysis of the number of full-time equivalent workers considering the equivalent conditions by various countries

Table S1. Analysis of the number of workers in the construction industry considering national data

Year	The number of workers in construction industry (number)					
	South Korea	Japan	China	Singapore	Mexico	The U.K.
2012	2,786,587	3,410,000	42,439,900	440,700	1,241,453	2,062,000
2013	2,566,832	3,420,000	44,727,400	479,300	1,211,501	2,126,000
2014	3,249,687	3,430,000	45,129,000	494,000	1,368,318	2,147,000
2015	3,358,813	3,420,000	50,665,800	501,200	1,416,283	2,217,000
2016	3,152,859	3,350,000	51,598,300	489,900	1,520,838	2,183,000
2017	3,046,523	3,380,000	55,028,900	451,800	1,563,708	2,313,000
2018	2,943,742	3,420,000	55,399,100	444,200	1,643,363	2,279,000
Min	2,566,832	3,350,000	42,439,900	440,700	1,211,501	2,062,000
Max	3,358,813	3,430,000	55,399,100	501,200	1,643,363	2,313,000
Average	3,015,006	3,404,286	49,284,057	471,586	1,423,638	2,189,571

Table S2. Analysis of the amount of construction revenue for calculation of full-time equivalent workers considering the equivalent conditions

Year	The amount of construction revenue ($)					
	South Korea	Japan	China	Singapore	Mexico	The U.K.
2012	274,499,434,000	418,222,290,000	2,041,366,950,000	23,378,664,000	21,951,486,800	120,365,460,000
2013	290,644,318,960	472,561,690,000	2,370,734,550,000	27,210,736,000	20,734,217,000	126,877,070,000
2014	296,285,694,560	490,691,960,000	2,462,519,245,500	29,455,396,000	21,248,571,900	135,549,170,000
2015	302,219,475,600	495,548,750,000	2,539,027,042,500	20,545,840,000	22,078,888,750	145,480,300,000
2016	328,042,232,480	497,694,390,000	2,706,546,943,500	20,066,888,000	22,667,675,550	148,344,970,000
2017	360,605,667,440	525,036,750,000	2,890,854,160,500	18,846,860,000	24,771,852,500	153,760,580,000
2018	362,626,036,200	529,580,230,000	3,156,859,867,500	23,206,752,000	26,130,568,750	160,083,130,000
Min	274,499,434,000	418,222,290,000	2,041,366,950,000	18,846,860,000	20,734,217,000	120,365,460,000
Max	362,626,036,200	529,580,230,000	3,156,859,867,500	29,455,396,000	26,130,568,750	160,083,130,000
Average	316,417,551,320	489,905,151,429	2,595,415,537,071	23,244,448,000	22,797,608,750	141,494,382,857

Table S3. Analysis of the amount of monthly wage of construction workers for calculation of full-time equivalent workers considering the equivalent conditions

**Year**	**The amount of monthly wage of construction workers ($)**					
	**South Korea**	**Japan**	**China**	**Singapore**	**Mexico**	**The U.K.**
2012	2,389	3,128	2,324	2,645	376	4,419
2013	2,562	3,201	2,819	2,816	371	4,340
2014	2,717	3,220	3,173	2,865	403	4,340
2015	2,905	3,314	3,247	3,001	426	4,551
2016	3,136	3,378	3,417	3,083	436	4,787
2017	3,301	3,336	3,589	3,216	468	4,814
2018	3,330	3,386	3,685	3,372	489	5,103
Min	2,389	3,128	2,324	2,645	376	4,419
Max	3,330	3,386	3,685	3,372	489	5,103
Average	2,906	3,281	3,179	3,000	424	4,622

Table S4. The labor ratio of South Korea for the construction industry

**Year**	**The labor ratio of South Korea**	
	**General construction work**	**Sub-contracting construction work**
2012	28%	32%
2013	28%	32%
2014	28%	32%
2015	27%	31%
2016	27%	31%
2017	27%	30%
2018	27%	30%
Application	The equivalent labor rate (27%) is applied to calculation of full-time equivalent workers.	

Table S5. Analysis of the number of full-time equivalent workers considering the equivalent conditions by various countries

Year	The number of full-time equivalent workers in construction industry (number)					
	**South Korea**	**Japan**	**China**	**Singapore**	**Mexico**	**The U.K.**
2012	2,584,749	3,008,072	19,767,905	198,888	1,313,237	612,849
2013	2,552,557	3,321,661	18,925,502	217,431	1,258,144	657,749
2014	2,544,008	3,428,322	17,462,867	231,309	1,186,040	702,706
2015	2,340,655	3,363,971	17,591,712	154,030	1,165,728	719,314
2016	2,353,520	3,315,467	17,819,735	146,471	1,168,571	697,208
2017	2,457,715	3,541,346	18,122,607	131,845	1,190,954	718,712
2018	2,449,890	3,518,785	19,273,433	154,844	1,203,434	705,837
Min	2,340,655	3,008,072	17,462,867	131,845	1,165,728	612,849
Max	2,584,749	3,541,346	19,767,905	231,309	1,313,237	719,314
Average	2,469,014	3,356,803	18,423,394	176,402	1,212,301	687,768
